# Value of Diffusion-Weighted MR Imaging for the Detection of Nephritis

**DOI:** 10.1155/2013/348105

**Published:** 2013-11-11

**Authors:** Benjamin Henninger, Miriam Reichert, Stefan Haneder, Stefan O. Schoenberg, Henrik J. Michaely

**Affiliations:** ^1^Institute of Clinical Radiology and Nuclear Medicine, University Medical Center Mannheim, Medical Faculty Mannheim, Heidelberg University, Theodor-Kutzer-Ufer 1-3, 68167 Mannheim, Germany; ^2^Department of Radiology, Innsbruck Medical University, 6020 Innsbruck, Austria

## Abstract

*Purpose*. To evaluate diffusion-weighted MR imaging (DWI-MRI) for the detection and assessment of infectious renal disease. *Materials and Methods*. Twenty-one patients with suspicious increased signal intensity of the kidneys on DWI sequences and corresponding ADC decrease were identified. Sixty patients without clinical signs of renal infection served as a control group. All patients were examined with the following sequences: EPI-DWI (0/400/800 s/mm^2^), T2w HASTE, and T1w VIBE after intravenous injection of Gd-chelate. Confirmation of renal infection was established on the basis of clinical criteria. T1w and T2w images were assessed and compared to DWI for the presence of altered signal, and the degree of the visibility of pathology was graded on an ordinal three-point scale. *Results*. In all 21 patients with positive DWI findings a renal infection could be confirmed. T2w imaging and contrast-enhanced T1w imaging displayed obvious pathologic signal in 3/21 (14%) and 11/19 (58%) patients and slightly pathologic signal in 17/21 (81%) and 7/19 (37%), respectively. The median visibility score of 2 for the DWI and the T1w images was significantly higher than the score of 1 for the T2w imaging, *P* = 0.0001 (DWI versus T2w) and *P* = 0.078 (T1w versus T2w). *Conclusion*. DWI of the kidneys seems to be highly sensitive for the detection of infections within the kidney.

## 1. Introduction

Infection of the kidneys is a common disease that might involve the renal parenchyma only (nephritis) or the parenchyma and the renal pelvis (pyelonephritis). When left untreated, the disease might lead to renal scarring with chronic renal failure and hypertension. The diagnosis is mainly based on the clinical presentation of the patients, who often suffer from fever and flank pain, and on laboratory workup and urine analysis. Radiology plays a minor role in the routine workup of these patients but is typically performed to establish the diagnosis in equivocal cases and in evaluating high-risk patients and to assess the extent of renal involvement including the presence of abscesses.

Ultrasound and contrast-enhanced CT studies represent the mainstay of radiologic exams in this context and typically show perinephric stranding, enlarged kidneys, and irregular contrast agent uptake of the affected kidneys [1]. If a renal calculus is suspected, low-dose CT is nowadays the imaging modality of choice [2]. Particularly in younger patients and patients with preexisting impaired renal function, the use of CT or contrast-enhanced CT is discouraged due to radiation exposure and the potential nephrotoxicity of iodinated contrast agents. A renal MR study using T2-weighted (T2w) sequences before and T1-weighted (T1w) sequences after Gd-chelate injection will show similar findings as a contrast-enhanced CT in inflammatory renal disease. In an animal model of acute pyelonephritis, almost identical results for sensitivity and specificity of CT exams (86.3%/87.5%) and MR exams (89.5%/87.5%) were found [3]. However, a complete renal MR exam is rather time consuming and comparable in its diagnostic accuracy to contrast-enhanced CT studies and is therefore barely performed in the acute setting of infectious renal disease.

Diffusion-weighted MR imaging (DWI) is rapidly gaining popularity for assessment of intra-abdominal oncologic and non oncologic pathologies [4–7]. Once a technique primarily used in neuroradiology, it is now gaining acceptance as a tool to further characterize alterations of random (Brownian) movement (i.e., diffusion) of water molecules within various lesions in the abdomen. The technique is in clinical use for determining pathology in the liver (degree of cirrhosis/fibrosis), kidneys (lesion characterization, renal failure), and other abdominal organs [4, 6–9].

However, the clinical value of DWI-MR for the detection and assessment of infectious diseases of the kidneys has only briefly been addressed in previous publications and review papers but has never been thoroughly investigated [10, 11]. Therefore, the aim of this study was to assess the value of DWI-MRI for the detection and assessment of infectious renal disease in comparison to standard MRI sequences in a case control study.

## 2. Material and Methods 

### 2.1. Patients

Diffusion-weighted imaging of the abdomen has been introduced as a standard imaging technique for all abdominal studies at our institution 24 months ago. After IRB approval, a retrospective analysis of the electronic radiologic medical records was performed to identify patients with suspicious, nontumorous findings of the kidneys on DWI sequences. As a nontumorous finding, a diffuse or patchy increase in the DWI source data with *b* = 800 s/mm² was considered. Patients with these positive medical record findings were reassessed by a radiologist with 12 years of experience in body imaging for the presence of the following imaging patterns: ADC value of the suspicious area and visibility of these findings in conventional T2w imaging and in postcontrast T1w imaging. This database search yielded a total of 21 patients (12 females, 9 males, mean age 50.0 ± 24.3 years, and age range 9–85 years). None of the patients suffered from hydronephrosis, or recurrent renal infections. Among the 21 patients were 4 patients with renal transplants. Two patients did not receive contrast agent. For the patients the presence of renal infection at the time of the MR imaging was established based on clinical test results (physical exam, urine analysis, laboratory findings, and course of disease) which were available in the electronic patient records for all patients.

A control group consisting of 60 patients without suspicious renal findings in DWI and without clinical proof of infectious renal disease was identified as well.

### 2.2. MR Imaging

Four different MR scanners were used in this study: two 32-channel 1.5 T MR systems (MAGNETOM Avanto 32 × 76 1.5 T; Siemens Healthcare; Erlangen, Germany), a 32-channel 3 T MR system (MAGNETOM Tim Trio 32 × 76 3 T; Siemens), and a 64-channel 3 T MR system (MAGNETOM Skyra; Siemens). All MR scanners were equipped with the same gradient systems. All studies were performed with the systems' standard anterior body matrix coils—six coil elements were included on the 1.5 T MR systems and the TimTrio, while an 18-element coil was used with the Skyra. Patients were positioned head first supine. A product EPI sequence was used for the acquisition of the DWI images during free respiration and before the contrast-agent administration. Images were obtained with routinely used *b* values of 0/400/800 s/mm², and automated ADC parameter maps were generated by the MR systems. Detailed sequence parameters are presented in Table 1. T2w imaging was performed with a two-dimensional coronal half-Fourier acquired single shot turbo spin echo sequence (HASTE) with 5 mm slice thickness and without fat saturation (TR/TE—1100/103 ms @ Avanto, 1100/98 ms @ TimTrio, 1400/80 ms @ Skyra, matrix 384 × 80% for all MR scanners, and parallel imaging acceleration factor 2 for all MR scanners). T1w imaging was performed with an axial three-dimensional volume interpolated breathhold exam (VIBE) sequence (TR/TE—5.09/1.87 ms @ Avanto, 3.42/1.4 ms @ TimTrio, 4.2/1.4 ms @ Skyra, slice thickness 3 mm, matrix 320 × 80%, and parallel imaging acceleration factor 2 for all three MR scanners) before and thrice after the injection of 0.1 mmol/kg body weight of a macrocyclic Gd-chelate (Gd-DOTA (Dotarem), Guerbet, Paris, France or Gadobutrol (1.0 M Gadovist), Bayer, Berlin, Germany) to obtain an arterial phase (20 s after the start of contrast agent injection), a portovenous phase (50 s after the start of contrast agent injection), and a delayed phase dataset (120 s after the start of the contrast agent injection). For the injection of the contrast, agent an MR compatible automated injector pump (Spectris Solaris, Medrad, Indianola, PA) was used at 1.5 T while at 3.0 T a different model (Spectris Solaris EP, Medrad) was employed.

### 2.3. Image Analysis

The ADC values were measured in the affected part of the kidney using a circular region of interest and were measured in the nonaffected part of kidney by a single radiologist with a 12-year experience in body MRI. The T2w-images and postcontrast T1w images of all patients were then assessed by another radiologist with 5 years of experience for the presence of altered T2w signal or T1w signal in any of the three phases of postcontrast administration. The degree of the visibility of pathology within the kidneys was graded on an ordinal three-point scale for the T1w images and the T2w images separately as follows: 0—normal kidney and no focal or diffuse alteration visible, 1—slightly visible focal or diffuse pathologic signal, and 2—obvious pathologic signal alterations. 

### 2.4. Statistical Analysis

Due to the retrospective and hypothesis-generating character of the analysis, no sample size estimation was performed. Statistical analyses were performed using dedicated statistical software (JMP 9.0, SAS Institute, Cary, North Carolina, USA). If appropriate the 95% confidence interval (95% CI) is provided as well. The Shapiro-Wilk *W* test was applied to identify normally distributed data. Descriptive statistics were performed using paired Wilcoxon-rank sum tests. *P* < 0.05 was considered to represent statistical significance.

## 3. Results

**Figure 1:**
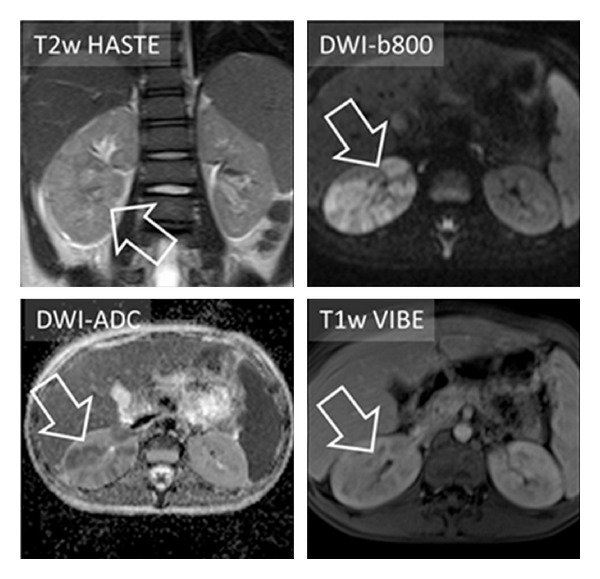
Nine-year-old male patient presenting with pain in the right flank. Laboratory examination revealed increased leukocytes (12.2 × 10^9^ cells/L) and CRP (153 mg/L). MRI scan with routine protocol for the abdomen was performed. It showed only slight signal alterations in T2 HASTE and T1w VIBE (postcontrast). DWI-b800 clearly depicted wedge-shaped, striated areas of high signal intensity in the right kidney with correlating low signal in DWI-ADC. The diagnosis of renal infection was finally done on the basis of clinical criteria.

**Figure 2:**
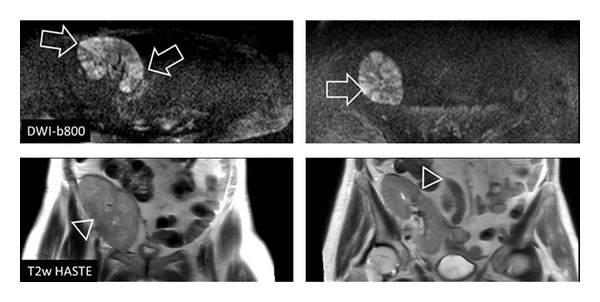
Fifty-eight-year-old female patient with kidney transplant in the right pelvic region. MRI scan showed multifocal, wedge-shaped signal alterations clearly in DWI-b800 (arrow) while T2w imaging could only depict slight pathologic signal (triangle). The diagnosis of nephritis was finally confirmed by urine analysis and laboratory findings.

**Figure 3:**
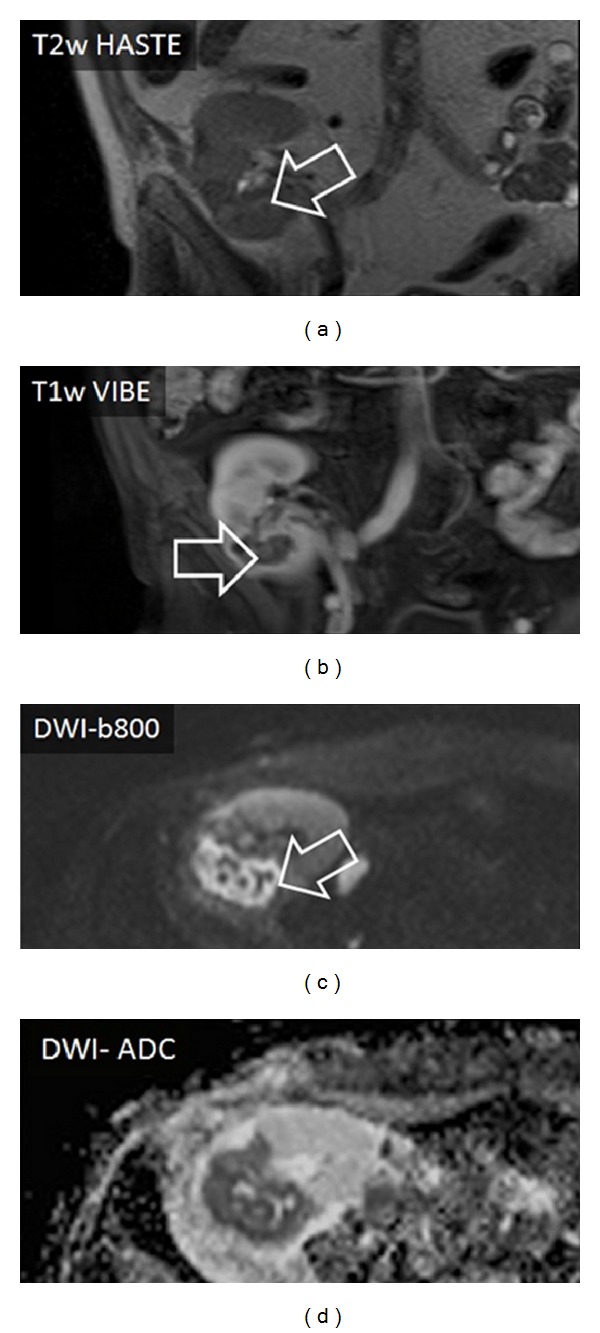
Sixty-five-year-old male patient with an abscess in the kidney transplant (right pelvic region). The abscess formation could be easily detected with DWI-b800 and correlating DWI-ADC present distinctive signal alterations. T2w imaging showed only marginal alterations whereas T1w imaging with contrast defined the abscess superiorly (arrow).

**Figure 4:**
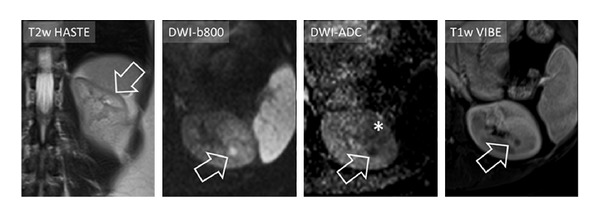
In a thirty-two-year-old female patient DWI-MRI was able to depict a small focal nephritis in the left kidney (arrow), which was surrounded by a patchy area of altered signal in DWI-b800 and DWI-ADC (asterisk in DWI-ADC). By using only T2w and T1w imaging the focal area of nephritis could easily be misinterpreted as, for example, a simple cyst.

**Figure 5:**
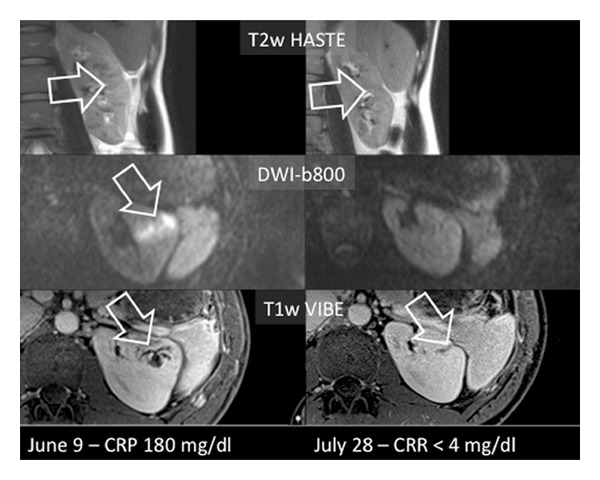
Pyelonephritis in a 31-year-old female patient of the left kidney who was imaged initially to assess the extent of renal involvement for possible operation and reimaged after 7 weeks of antibiotic therapy. Left column: a clear infectious focus can be easily seen on the DWI images and on the venous T1w images and the signal alterations of the kidney appear diffuse on T2w imaging. Right column: after successful antibiotic therapy with normal CRP value, no pathologic signal changes can be seen on the DWI images. The T1w and T2w images show moderate scarring of the formerly affected parenchyma (arrows).

In all 21 patients with DWI positive findings in the kidneys, a renal infection was confirmed based on clinical tests while none of the 60 control patients revealed clinical signs of infection (Table 2). A detailed representation of the patients' clinical findings is provided in Table 3. Typical pathologic imaging patterns in the DWI images were wedge-shaped striated multifocal areas of high signal intensity in the DWI source data with *b* = 800 s/mm² (Figures 1 and 2). Diffuse signal intensity increase throughout parts of a kidney which was not wedge-shaped was encountered only in two cases. One patient had an abscess of the kidney which was clearly defined on DWI (Figure 3). Even subtle infectious foci could be seen and characterized using DWI imaging while being nonspecific on T2w and T1w imaging (Figure 4). In one patient who was imaged during an acute pyelonephritis and after successful antibiotic therapy, the pathologic signal in the DWI images has completely vanished after therapy (Figure 5). In all 60 control patients, no pathologic alteration of the renal DWI signal was seen. The infected areas within the kidneys DWI revealed decreased mean apparent diffusion coefficients of 1.1 ± 0.3 × 10^−3^ mm²/s at 3 T and 1.2 ± 0.3 × 10^−3^ mm²/s at 1.5 T which was significantly lower than the ADC of nonaffected renal tissue of 1.7 ± 0.2 × 10^−3^ mm²/s at 3 T and 1.9 ± 0.2 × 10^−3^ mm²/s at 1.5 T, respectively, (*P* < 0.0001). In the control group, the ADC of the kidneys was 1.9 ± 0.1 × 10^−3^ mm²/s at 1.5 T and 1.8 ± 0.1 × 10^−3^ mm²/s at 3 T hence not showing a significant deviation from the healthy areas of the sick patients' kidneys. The DWI images of the diseased kidneys presented an obvious pathologic signal in 18/21 (86%) patients while demonstrating a slightly pathologic signal in 3/21 (14%) patients. Contrast-enhanced T1w imaging displayed obvious pathologic signal in 11/19 (58%) patients, slightly pathologic signal in 7/19 (37%) patients and was negative in 1/19 (5%) patient. For two patients who declined contrast agent injection no data exist. T2w imaging presented obvious pathologic signal in 3/21 (14%) patients, slightly pathologic signal in 17/21 (81%) patients, and no pathologic changes in 1/21 (4%) patient. The median visibility score of 2 for the DWI imaging and the T1w images was significantly higher than the median visibility score of 1 for the T2w-imaging (*P* = 0.0001 and *P* = 0.078). No significant differences were found between the visibility scores of T1w imaging and DWI. 

## 4. Discussion

The results of this study are very encouraging as they suggest that non-contrast-enhanced DWI imaging of the kidneys seems to be more sensitive than conventional MR imaging with T2w and postcontrast T1w sequences. The exact clinical value of DWI-MR for the detection and assessment of infectious diseases has not yet been exactly investigated. Solely two review papers [10, 11] and a single case report on an infected cyst in a patient with polycystic kidney disease [12] addressed the value of DWI in infectious renal disease. The diffusion restriction seen on DWI is thought to be a consequence of an increased cellular density caused by accumulation of leukocytes in the infected areas of the kidneys while in case of renal abscesses the diffusion restriction is caused by the pus within the cavity. Blunt renal abscesses do not represent a diagnostic challenge and can be easily recognized with ultrasound (US) or CT. Smaller foci of infection or diffuse disease only affecting parts of the kidneys are, however, harder to detect with CT and US. In an animal study on the detection of acute pyelonephritis, CT and MR imaging demonstrated almost identical accuracies with sensitivities and specificities of >86% and 87%, respectively, while Doppler ultrasound achieved a disappointing sensitivity and specificity of 74.3% and 56.7% only [3]. 

Technical developments have increased the capabilities of ultrasound since then. A recent prospective study on detection of acute pyelonephritis with contrast-enhanced ultrasound of renal transplants found a sensitivity and specificity of 95% and 100% compared to contrast-enhanced T1w-MR imaging as standard of reference with excellent inter-modality agreement of *K* = 0.92 [13]. However, no use was made of DWI-MRI in this study. Looking closer at our data it seems that particularly in those patients with minor foci of infection or without abscess formation, the sensitivity of DWI imaging differs most from T1w and T2w imaging and clearly demonstrates foci of infection which can neither be seen nor be characterized due to too small size. A noticeable characteristic of DWI is that in the source data with high *b* values (i.e., *b* = 800 s mm²) even the smallest foci of infection are displayed with high lesion-to-background contrast. This high conspicuity of inflammatory changes in combination with the assumed high sensitivity could foster the use of DWI as a primary tool for workup of complicated patients or patients with impaired renal function. As CT is increasingly often recognized as major source of radiation exposure for the general population, some experts recommend “to replace CT use, when practical, with other options, such as magnetic resonance imaging (MRI)” [14]. In the setting of infectious renal disease where many nononcologic patients and younger patients are being examined this is of even higher importance. In pediatric patients the comparable radiation dose is on average 24% higher compared to the already high dose in abdominal studies in adults [15]. Especially for pediatric patients DWI-MRI of the kidneys seems to be a perfect match combining a radiation-free examination of the abdomen with high robustness to motion as it can be acquired during continuous breathing. When applying DWI imaging to the kidneys to identify infectious foci, some caveats have to be considered. First, it is well known that chronic hydronephrosis and resulting renal fibrosis might lead to decreased ADC values of the kidneys [16, 17]. Also transplant kidneys with acute deterioration of function were found to have lower ADC values in one study than transplants with normal function [18]. Unlike the diffuse diffusion restriction seen in the latter two mentioned clinical settings, findings of infectious renal disease are often patchy and irregular and barely ever affect the entire kidney homogenously. Also, the usual clinical presentation of these patients will differ significantly. 

The diffusion restriction in DWI can be absolutely quantified by means of the apparent diffusion coefficient (ADC). This is routinely done in oncologic imaging in order to investigate the malignant potential of the tumor (high grade tumors typically reveal lower ADC values) and to assess the effects of therapy on malignant lesions (typically ADC will increase with response to therapy) [7]. In this study ADC values of infected renal tissue were significantly lower than the ADC values of healthy renal tissue without significant differences between the two employed field strengths. A safe differentiation of infection from malignant tumors with atypical infiltrative growth pattern such as transitional cell carcinomas hence does not seem feasible, based on the ADC values only.

CT of infectious renal disease is still the mainstay of imaging in radiology. There is a wealth of knowledge on CT appearances of various rare renal disease conditions such as papillary necrosis [19] or emphysematous pyelonephritis [20] and xanthogranulomatous pyelonephritis. While these conditions might also be detected on MRI [21], the detectability of air and calcifications is much higher in CT. Particularly the potential to display renal calculi with high accuracy [22] and at low radiation dose [2] triggers a large amount of CT exams of the urogenital system. Apart from availability which is still limited for MRI at many sites, the clinical questions, “urolithiasis” and “gas-forming infection,” will further require a CT study to be performed. 

### 4.1. Study Limitations

This study has some well-recognized limitations: first, as a retrospective study, only those patients with DWI positive signal were included while, in a prospective study setting, the clinical presentation would trigger the imaging. Therefore a bias favoring the sensitivity of DWI of the kidneys cannot be ruled out. However, all patients with pathologic findings on DWI finally suffered from pyelonephritis/nephritis. To further mitigate the potential bias of including only patients with a pathologic DWI signal, a control group was included of which none of the patients demonstrated a pathologic signal on DWI imaging. Second, no simultaneous CT imaging or ultrasound was available for comparison. A comparison to another imaging modality as standard of reference would have further increased the validity of this study. This comparison should be done under controlled conditions in a clinical trial.

### 4.2. Conclusion

Based on this hypothesis-generating study DWI of the kidneys seems to be highly sensitive for the detection of focal or diffuse infections within the kidney. Compared to conventional T2w and postcontrast T1w imaging, DWI appears to facilitate the detection of infected areas without contrast administration. Further, prospective studies are warranted to further investigate into DWI in infectious renal disease.

## Figures and Tables

**Table 1:** Sequence parameters of the used DWI sequences at the different scanner platforms.

	1.5 T Avanto	3.0 T TimTrio	3.0 T Skyra
TR/TE [ms]	5600/75	6000/76	6400/63
Sequence type	EPI-SE	EPI-SE	EPI-SE
FOV [mm × mm]	380 × 308	380 × 308	380 × 308
Matrix	192 × 156	192 × 156	192 × 156
Slice thickness [mm]	6	5	5
Interslice gap [mm]	0	0	0
Spatial resolution [mm³]	2.0 × 2.0 × 6.0	2.0 × 2.0 × 5.0	2.0 × 2.0 × 5.0
Number slices	32	33	35
*b* values [s/mm²]	0, 400, 800	0, 400, 800	0, 400, 800
Parallel imaging	GRAPPA 2	GRAPPA 2	GRAPPA 2
Acquisition time [min]	4:30	5:06	4:46
Respiratory control	Free breathing	Free breathing	Free breathing
Fat suppression	SPAIR	SPAIR	SPAIR
Averages	4	4	3
Bandwidth [Hz/px]	1736	1736	1736

**Table 2:** Overview of the patient and controls.

	Patients		Controls	
Field strength	1.5 T	3.0 T	1.5 T	3.0 T
Gender	7 m/5 f	2 m/7 f	22 m/13 f	12 m/13 f
Mean age [years]	47.9	45.9	54.9	48.3
Mean ADC kidney [mm²/s] ± SD	1866 ± 177	1710 ± 237	1872 ± 110	1786 ± 117
Mean ADC inflammatory focus [mm²/s] ± SD	1255 ± 263	1146 ± 261	n/a	n/a

ADC: apparent diffusion coefficient, SD: standard deviation.

**Table 3:** Patient characteristics.

Sex	Age	Final diagnosis	Urin-stix	Leukocytes	CRP (mg/L)	T2w	Postcontrast T1w	DWI-b800
M	65	Nephritis, abscess	+	4.0	<2.9	++	++	++
W	80	Pyogenic nephritis	+	4.3	70	+	++	++
M	9	Nephritis	Not done	12.2	153	+	++	++
W	58	Pyelonephritis	+	10.0	46	+	+	++
M	62	Pyelonephritis	+	14.3	162	+	++	+
M	76	Pyelonephritis	+	9.0	16.9	+	+	+
W	61	Nephritis	+	13.3	92.5	+	++	++
W	73	Nephritis	+	4.4	27	+	++	++
W	24	Nephritis	+	28.3	174	+	++	++
W	16	Pyelonephritis	+	11.3	156	+	Not done	++
W	57	Pyogenic nephritis	Not done	12.7	54	++	++	++
W	31	Pyelonephritis	+	18.4	180	++	++	++
W	42	*E. coli* sepsis	+	13.8	42.2	+	+	++
M	32	Focal nephritis	+	Not done	Not done	−	−	++
W	17	Pyelonephritis	−	17.5	119	+	+	++
W	18	Pyelonephritis	Not done	21.1	73.2	+	+	+
M	85	Pyelonephritis	+	5.8	92.5	+	++	++
W	40	Pyelonephritis	+	11.5	224	+	++	++
M	65	Pyelonephritis	+	19.4	160	+	+	++
M	56	Nephritis	+	6.5	4.2	+	+	++
M	84	Nephritis	+	4.9	42.2	+	Not done	++
